# A Modular Polysaccharide Platform With Tannic Acid‐Driven van der Waals Lock‐In for Programmable Protein Delivery

**DOI:** 10.1002/smmd.70061

**Published:** 2026-10-29

**Authors:** Weibin Wu, Zheng Peng, Zehua Liu, Yi Yu, Danyang Chen, Jiarou Zhong, Fuhua Zhang, Yuting Fan, Shaogui He, Ruolan Ye, Shan Jiang, Yun Hong, Caisheng Wu, Qiang Xie

**Affiliations:** ^1^ Department of Cardiology The First Affiliated Hospital of Xiamen University School of Medicine Xiamen University Xiamen China; ^2^ Division of Pharmaceutical Chemistry and Technology Faculty of Pharmacy University of Helsinki Helsinki Finland; ^3^ Fujian Provincial Key Laboratory of Innovative Drug Target Research and State Key Laboratory of Cell Stress Biology School of Pharmaceutical Sciences Xiamen University Xiamen Fujian China; ^4^ Xiamen Key Laboratory for Clinical Efficacy and Evidence‐Based Research of Traditional Chinese Medicine Xiamen University Xiamen China; ^5^ State Key Laboratory of Vaccines for Infectious Diseases Xiang An Biomedicine Laboratory Xiamen Fujian China; ^6^ School of Medicine Xiamen University Xiamen Fujian China

**Keywords:** anticoagulant, Hirudin, polysaccharide, protein delivery, tannic acid

## Abstract

Protein delivery systems face persistent challenges, including low encapsulation efficiency, limited targeting ability, and lack of generalizability. Here, we propose a modular and programmable polysaccharide‐based platform, enabling efficient encapsulation of a wide range of proteins, and tunable targeting across different cell types. The stepwise assembly applies tannic acid (TA) for protein surface stabilization. This rewires the encapsulation mechanism from unstable electrostatic binding to robust van der Waals‐driven protein lock‐in. The TA‐protein complex can be encapsulated in an interchangeable polysaccharide nano‐framework, exploiting the lectin‐glycan recognition pattern to direct cell‐ and disease‐specific targeting. The TA‐Polysaccharide Encapsulation (TAPE) system robustly achieves > 85% encapsulation efficiency (EE) across a range of proteins, and tailoring the polysaccharide choice enables programmable targeting to distinct cell types. As a proof of concept, hirudin‐loaded TAPE achieved targeted anticoagulant treatment in vivo. Overall, our work positions TAPE as a simple and generalizable platform that overcomes fundamental barriers in protein delivery, offering wide translational potential for protein‐based therapies.

## Key Points

- High encapsulation efficiency across diverse proteins with robust reproducibility;
- Clear mechanistic basis for improved encapsulation via in silico exploration;
- Programmable cell targeting through interchangeable polysaccharide selection;
- Simple and streamlined fabrication process without extra chemical modification;
- Validated application and therapeutic advances in clinically relevant thrombosis models.

## Graphical abstract

Our study establishes a generalizable strategy for protein delivery that integrates efficient encapsulation with programmable targeting. By linking interaction mechanisms with delivery performance, it provides a practical route for developing protein therapeutics and may inform the design of broader nanomedicine systems.

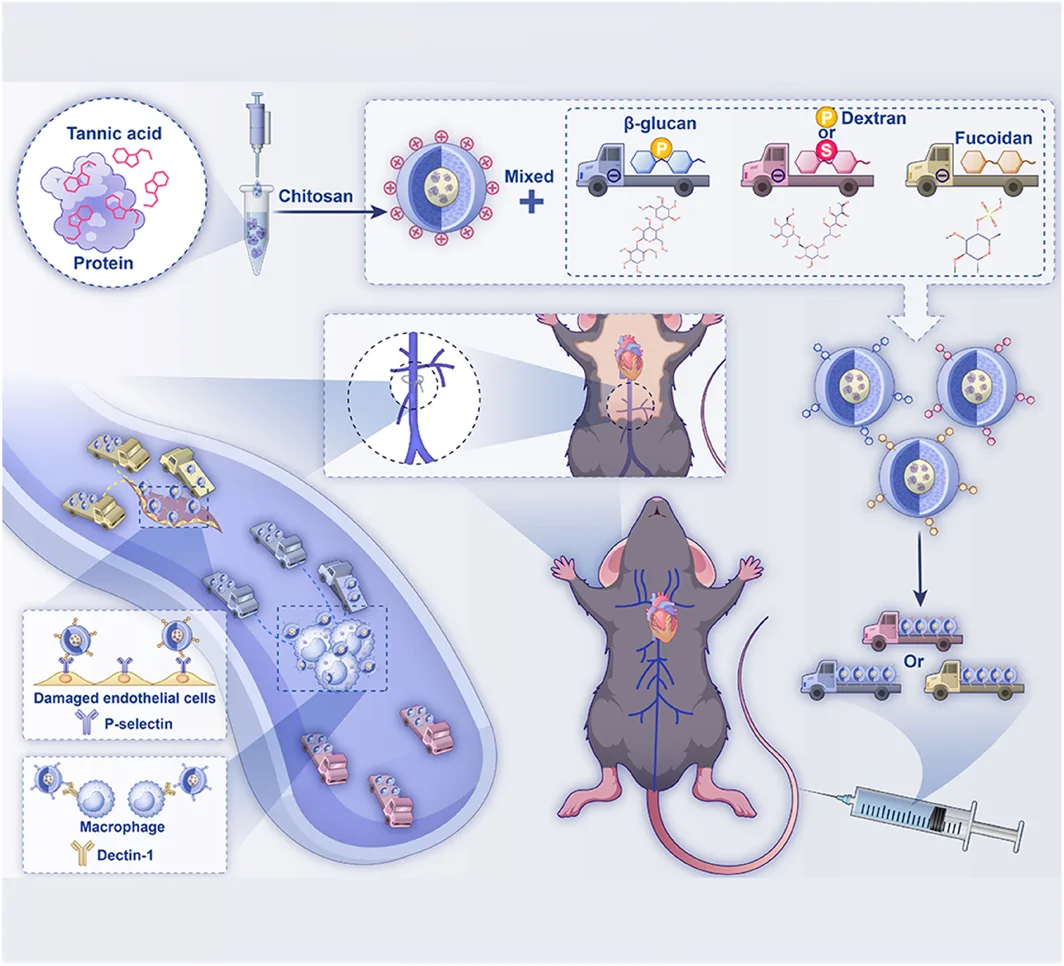

## 1 Introduction

Protein‐based therapeutics have surged over the past decade, proving to be among the most precise and effective drugs to tackle a wide range of diseases [1, 2]. However, the fragile and hydrophilic nature of the majority of proteins restricts their ability to cross biological barriers, making protected and targeted delivery essential to unlock their full therapeutic potential [3, 4]. In combination with their expensive cost, effective protein delivery systems therefore demand both robust and efficient encapsulation to preserve and protect their activity, while achieving precise targeting to enrich the loaded protein at sites of action.

Nanotechnology offers a promising avenue, leveraging its capacity to traverse biological barriers and provide tunable surfaces for precise targeting [5, 6]. One common method for protein nano‐encapsulation is by electrostatic interaction with cationic blocks (e.g., polymers or lipids) [7]. This approach offers advantages in simplicity and convenience, but owing to the inherently dynamic nature of electrostatic interactions and the hydrophilic nature of loaded protein, such systems frequently display a restrained encapsulation efficiency (EE) and burst release in biological environments [8, 9]. Polysaccharide‐based protein carriers have evolved from early ionically assembled systems toward increasingly functional nanocarriers, yet burst release remains a recurrent limitation of electrostatically assembled formulations. For example, in chitosan based nanoparticles (NPs), proteins associated with or located near the particle surface can rapidly desorb or diffuse into the surrounding medium, and optimization of formulation parameters can modulate, but does not fully overcome, this initial burst effect [10]. Alternative approaches have focused on increasing protein processability through chemical modification or by crystallizing proteins in biphasic solvents. While showing improved encapsulation efficiency, these processes bring risks of protein denaturation and add significant complexity to manufacturing [11]. Moreover, common approaches typically need to be tailored for each individual protein, limiting their general applicability. Therefore, an ideal system should combine high EE with a simple process and be broadly transferable across diverse protein therapeutics.

Beyond efficient encapsulation, precise targeting has become another central requirement for maximizing the protein therapeutic efficacy [12]. Ligand‐modification represents one of the most widely used targeting strategies [13, 14]. However, the preparation process typically requires extensive chemical/physical coupling and purification, which brings translational hurdles including protein premature release, high production cost and batch‐to‐batch variation [15, 16, 17]. Recent biomaterial platforms have introduced programmability through spatial organization and material‐responsive design. Multifunctional protein delivery systems illustrate how structural and compositional organization can encode distinct biological functions, while protein‐focused systems based on polyphenol‐mediated supramolecular interactions or engineered contractile injection machinery enable programmable control over protein loading, release, or target specificity [18, 19, 20]. As an alternative, recent work, including ours, has introduced a simplified design principle by tailoring the choice of polysaccharides as building blocks for NPs [21, 22, 23]. The targeting rationale underlies the intrinsic recognition of specific glycans by disease‐associated lectins, therefore enabling targeted delivery without additional modification [24, 25, 26].

These considerations motivated us to decouple protein stabilization from biological targeting, so that a common cargo‐retention mechanism could be maintained while the targeting component is independently exchanged. Upon such consideration, we herein introduce a modular and programmable platform designed to overcome fundamental barriers in protein delivery, termed as Tannic Acid‐Polysaccharide Encapsulation (TAPE) system. The system is constructed in a hierarchical manner, where tannic acid (TA) is first introduced to stabilize protein surfaces, therefore shifting encapsulation from electrostatic interaction to robust van der Waals lock‐in [27, 28, 29, 30]. The stabilized TA‐protein complexes are sequentially incorporated into a polysaccharide nano‐framework (Figure 1), assembled from cationic chitosan (CS) and interchangeable anionic polysaccharides (APs) [31, 32]. Compared to the direct protein encapsulation by CS and APs, TAPE robustly promotes the EE to > 85% across various proteins, and the molecular mechanism for this polyphenol‐guided encapsulation reprograming is elaborated in silico. Furthermore, by tailoring the selection of APs, the nano‐framework can be programmed to exploit lectin recognition for cell‐ and disease‐specific targeting. As a proof of concept, we tested four representative APs, including phosphorylated α‐dextran (PD), sulfated α‐dextran (SD), phosphorylated β‐glucan (PG), and fucoidan (FUD), to fabricate distinct TAPE systems. By tailoring the AP component, the systems demonstrated tunable targeting, enabling preferential delivery to lesional macrophages, endothelial cells, or reduced cellular interaction for prolonged circulation. Lastly, we encapsulated hirudin within FUD‐based TAPE and achieved targeted anticoagulant efficacy in vivo. Together, these results establish TAPE as a simple, generalizable and translational strategy for protein therapeutics.

**FIGURE 1:**
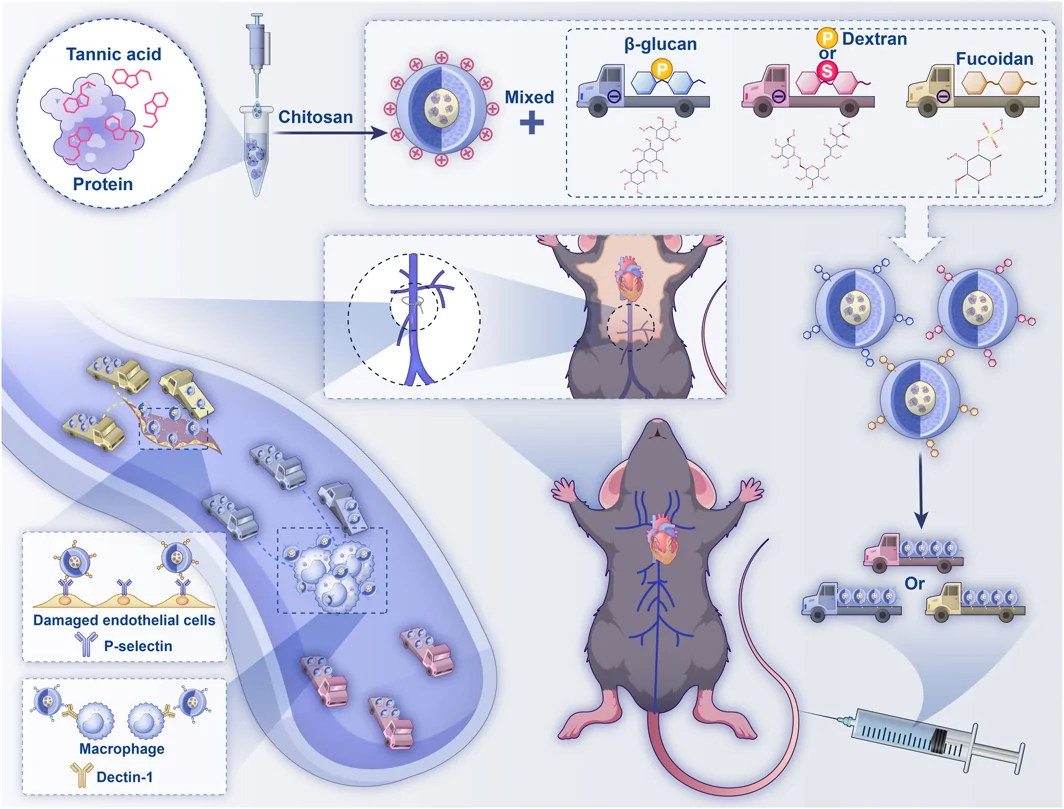
Schematic illustration of targeted protein delivery realized by polysaccharide‐framework.

## 2 Results

### 2.1 Interchangeable Polysaccharide Frameworks Enable Stable NPs Assembly

Followed by our design criteria, the assembly of polysaccharide NPs fundamentally determines the physiochemical properties of protein delivery systems such as size, polydispersity (PDI) and stability [33, 34]. Therefore, our first step is to systematically compare different polysaccharide pairings to define the optimal parameters for NP production. Briefly, CS was immobilized as a cationic part, which was further co‐assembled with different APs, including PD, SD, PG and FUD, in an aqueous solution. The relative weight ratios for the concentrations of APs and CS (AP/CS) were set to 0.25, 0.5, 1, 2.5, and 5 by mixing 1:1 volumetric ratios while maintaining a constant concentration of CS (Figure 2a–d). All 4 pairings yielded stable NPs, underscoring the interchangeable nature of the current strategy. With increasing AP ratios, the surface ζ‐potential gradually shifted from positive to negative values, indicating gradual surface exposure of APs. In our sequential experiments, we employed formulations that yielded the smallest size and PDI, while also exhibiting a negative charge to ensure that the corresponding APs remained exposed on the NPs surface, serving as functional modules for subsequent lectin recognition and targeting. Transmission electron microscopy (TEM) images (Figure 2e) were acquired for representative formulations (PG/CS = 5, PD/CS = 5, SD/CS = 2.5, and FUD/CS = 2.5, weight ratio). The observed morphologies were consistent with the dynamic light scattering (DLS) measurements, confirming the formation of well‐dispersed and uniformly sized NPs.

**FIGURE 2:**
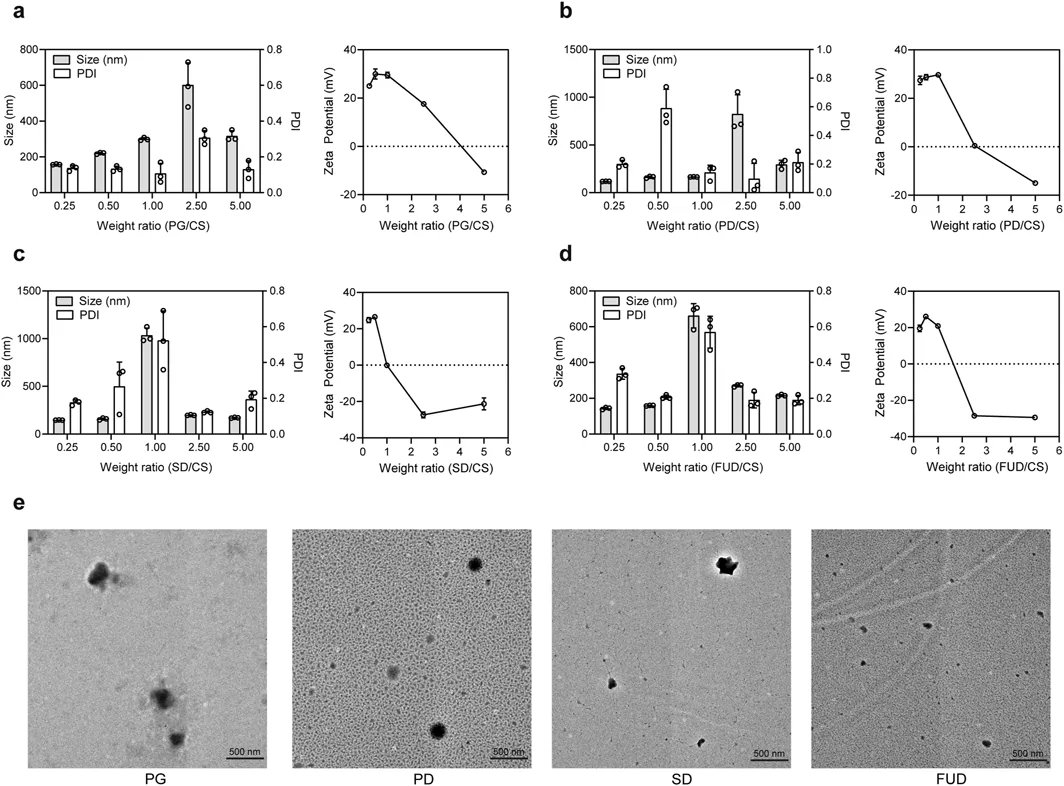
Characterization of different polysaccharide NPs. (a) Particle size, PDI and Zeta potential of different weight ratios for the concentrations of PG/CS. (b) Particle size, PDI and Zeta potential of different weight ratios for the concentrations of PD/CS. (c) Particle size, PDI and Zeta potential of different weight ratios for the concentrations of SD/CS. (d) Particle size, PDI and Zeta potential of different weight ratios for the concentrations of FUD/CS. (e) TEM of different polysaccharide NPs. Data are presented as mean ± standard deviation, *n* = 3. CS, cationic chitosan; FUD, fucoidan; PD, phosphorylated α‐dextran; PG, phosphorylated β‐glucan; SD, sulfated α‐dextran.

### 2.2 The Incorporation of TA Robustly Enhances the Protein Encapsulation Efficiency

Having established the polysaccharide framework, we next evaluated its potential for direct protein encapsulation. Hirudin (HRD) was selected as a model protein owing to its clinical relevance and acidic isoelectric point (pI 3.5–4) [35, 36]. To promote electrostatic interaction with the cationic CS backbone, formulations were prepared at pH 5.5, where HRD carries a net negative charge. For each AP‐based NPs, we conducted orthogonal experiments considering 3 formulation factors: CS concentration, HRD concentration, and AP concentration [37]. Across all formulations, the encapsulation efficiency (EE) of HRD remained below 60%, with marked variability between different APs and considerable batch‐to‐batch deviation (Supporting Information S1: Table S1). These results underscore the need for an improved strategy to robustly enhance protein loading.

Polyphenolic compounds have been reported to enhance the processability of proteins by stabilizing their surface interactions [38, 39, 40]. Inspired by this principle, we hypothesized that tannic acid (TA) could be used to precondition proteins, thereby improving both the efficiency and robustness of encapsulation. To test this, we first examined the TA‐HRD interactions. Turbidity measurements at 600 nm revealed an increase when the TA/HRD molar ratio exceeded 10, potentially suggesting complex formation (Supporting Information S1: Figure S1a). Meanwhile, we mixed FITC‐BSA with TA at varying molar ratios and measured the fluorescence intensity of the solution system across the full‐wavelength range under 488 nm excitation light. Notably, as the TA concentration increased, the fluorescence intensity of the system gradually diminished, suggesting that TA and FITC‐BSA had formed a complex. In this bound state, the fluorescence of the protein was shielded (Supporting Information S1: Figure S1b). Overall, these results collectively indicate the assembly of TA‐HRD complexes.

We next assessed whether the complexes could improve protein encapsulation in polysaccharide NPs. The NPs were prepared by dissolving CS (1 mg/mL^−1^), TA and BSA (0.2 mg/mL^−1^) in 0.1% acetic acid, and the final concentration of TA was tuned to achieve defined BSA/TA molar ratios (10, 20, 40, 80 and 100). The resulting solution was then mixed with different AP solutions at equal volumes, using AP/CS ratios optimized in our earlier screening to yield TA‐Polysaccharide Encapsulation (TAPE) system. The results suggested that regardless of the APs employed, different formulations robustly achieved EE exceeding 80% and remained highly reproducible, in sharp contrast to the low and variable EE observed with TA‐absent formulations (Figure 3a). Based on these findings, we subsequently established a protein‐TA molar ratio of 1:20 as the optimal formulation for TAPE. To further assess generalizability and versatility, we tested additional proteins, including hirudin, ovalbumin (OVA) and insulin. All formulations consistently achieved high EE ranging from 82% to 97%, with the lowest value observed in hirudin loaded in FUD based TAPE (FUD‐TAPE‐HRD) still above 80% (Figure 3b). The protein loading capacity (LC) of different TAPEs ranged from 3% to 10% (Supporting Information S1: Figure S2).

**FIGURE 3:**
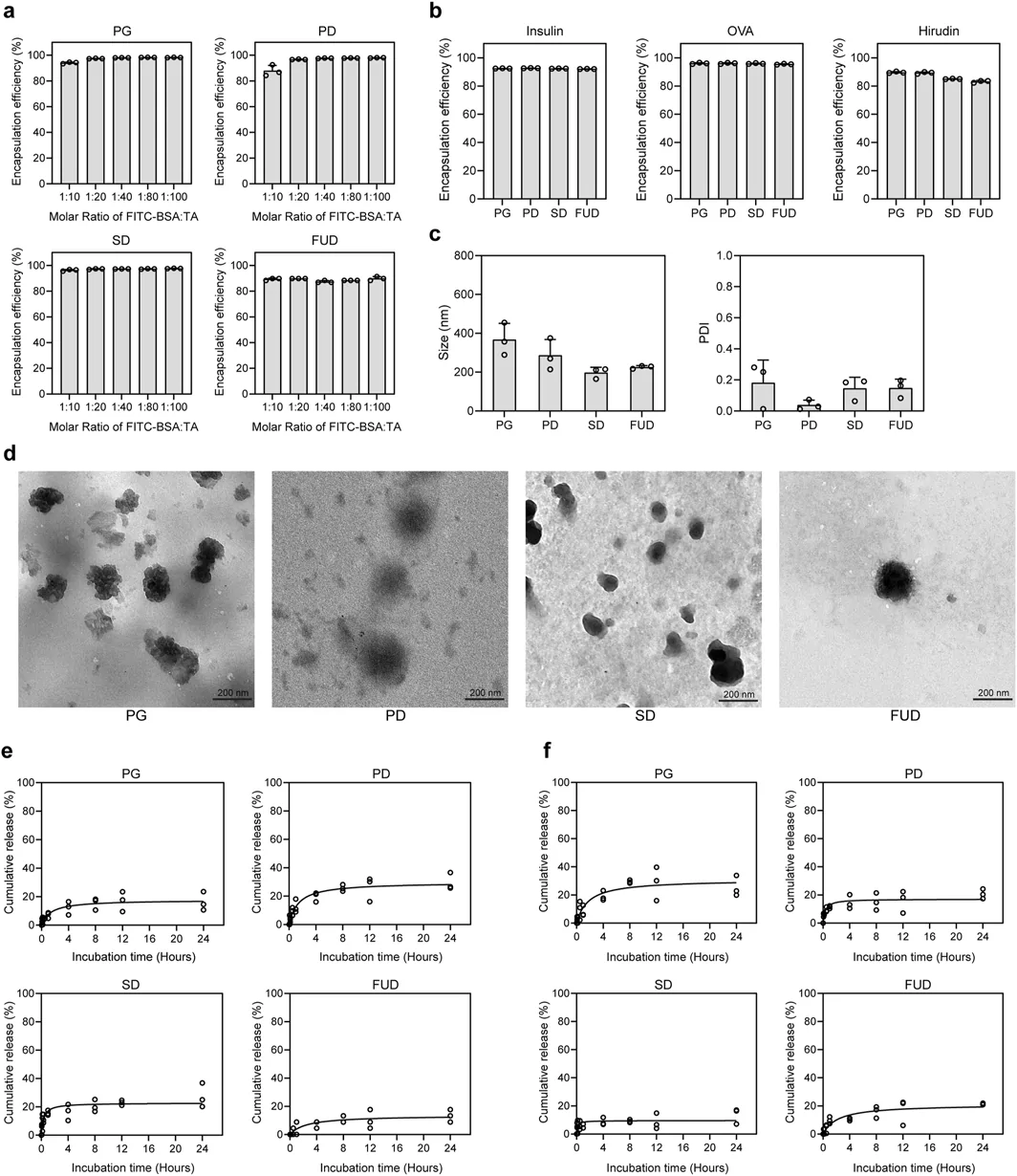
The physical characterizations of TAPE system and its release profile. (a) The encapsulation efficiencies of different TAPE systems at different molar ratios of FITC‐BSA/TA. (b) The EE of insulin, ovalbumin (OVA), and hirudin by different TAPE systems. (c) The particle size and PDI of TAPE systems loaded with hirudin. (d) The TEM images of the TAPE systems loaded with hirudin. (e) The protein release profiles of different TAPE systems under in vitro simulated physiological conditions. (f) The protein release profiles of different TAPE systems under acidic conditions. Data are presented as mean ± standard deviation, *n* = 3. CS, cationic chitosan; FUD, fucoidan; PD, phosphorylated α‐dextran; PG, phosphorylated β‐glucan; SD, sulfated α‐dextran.

Subsequently, the particle size and PDI of different TAPE systems loading hirudin were measured. Although the particle size was marginally larger than prior to protein loading, it remained below 500 nm (Figure 3c). To further observe the morphological changes upon protein encapsulation, TEM images of different TAPE systems loading hirudin were obtained (Figure 3d), and the results confirmed comparable particle sizes with DLS results. Finally, given that freeze‐drying is a widely used strategy for protein‐based products, we evaluated the performance of TAPE under lyophilization [41]. We screened a panel of cryoprotectants (mannitol, glycine, maltose) at 1%, 2%, 3% (w/v) and identified 2% glycine as the most effective, preserving particle size and PDI with minimal variation after lyophilization and reconstitution (Supporting Information S1: Figure S3). Together, these results demonstrate that TAPE enables robust and high‐efficiency encapsulation across diverse proteins while maintaining favorable particle stability and compatibility with lyophilization.

### 2.3 TAPE Enables Sustained Protein Release While Preserving Protein Bioactivity

Subsequently, we assessed the protein release from different TAPE systems. BSA was used as the model protein and was labeled with FITC [42, 43, 44]. The release profiles of FITC‐BSA from different TAPEs were determined in phosphate buffer saline (PBS) at 37°C over time. After 24 h of incubation, the protein release percentage for PG, PD, SD and FUD incorporated TAPE was 16.30 ± 5.39%, 29.66 ± 4.96%, 27.36 ± 7.00% and 13.30 ± 3.58% separately (Figure 3e), indicating a controlled and sustained protein release profile from different TAPE systems under physiological environment. Subsequently, we adjusted the environment to 37°C and pH 4 PBS buffer and re‐measured the release profile of FITC‐BSA. After 24 h of incubation, the protein release percentage for PD, PG, SD and FUD incorporated TAPE was 25.54 ± 6.05%, 20.69 ± 2.85%, 13.56 ± 4.55% and 21.50 ± 0.45% separately. These results demonstrate that TAPE maintains a controllable and stable protein release profile under acidic conditions (Figure 3f). In particular, we subjected the TAPE to repeated cycles of centrifugation and resuspension, thereby dynamically altering the release conditions to verify complete protein release. By the 5th cycle, nearly all FITC‐BSA in the TAPE had been fully released, confirming that the TAPE enables sustained protein release without inducing irreversible binding (Supporting Information S1: Figure S4).

Given that efficient encapsulation and sustained release are only therapeutically meaningful when protein function is preserved, we next examined whether TAPE formulation and subsequent release affected protein bioactivity. Insulin was selected as a functionally quantifiable model protein, and its hypoglycemic activity was evaluated in a type I diabetic mouse model [45]. Free insulin induced a rapid reduction in blood glucose, whereas FUD‐TAPE‐INS exhibited a slower onset of glucose lowering, consistent with the gradual availability of encapsulated insulin from the matrix. Importantly, insulin recovered from FUD‐TAPE after the release procedure retained a pronounced hypoglycemic effect, while blank FUD‐TAPE showed no glucose‐lowering activity (Supporting Information S1: Table S3). These results provide functional evidence that insulin remains pharmacologically active after both TAPE encapsulation and subsequent release.

### 2.4 Mechanistic Insights Into TA‐Assisted Protein Encapsulation

To elucidate the molecular mechanism underlying the TA‐mediated encapsulation enhancement, molecular dynamics (MD) simulations were performed to dissect the interaction modes among HRD, CS, TA, and APs during formulation processing [46, 47]. We first examined the encapsulation behavior of HRD in the absence of TA. Initiated by the complexation between HRD and CS, HRD associated with CS within 20 ns (Figure 4a), and the binding energy was primarily based on electrostatic attraction (Coulombic potential, Figure 4b). However, the Coulombic potential exhibited large oscillations over time, accompanied by pronounced root mean square deviation (RMSD) fluctuations (Figure 4c). When PG molecules were sequentially introduced to mimic the NPs assembly (Figure 4d), the Coulombic potential (Figure 4e) as well as the RMSD (Figure 4f) showed temporal enhancement, reflecting that the competitive interaction between PG and CS affected the HRD‐CS complex stability.

**FIGURE 4:**
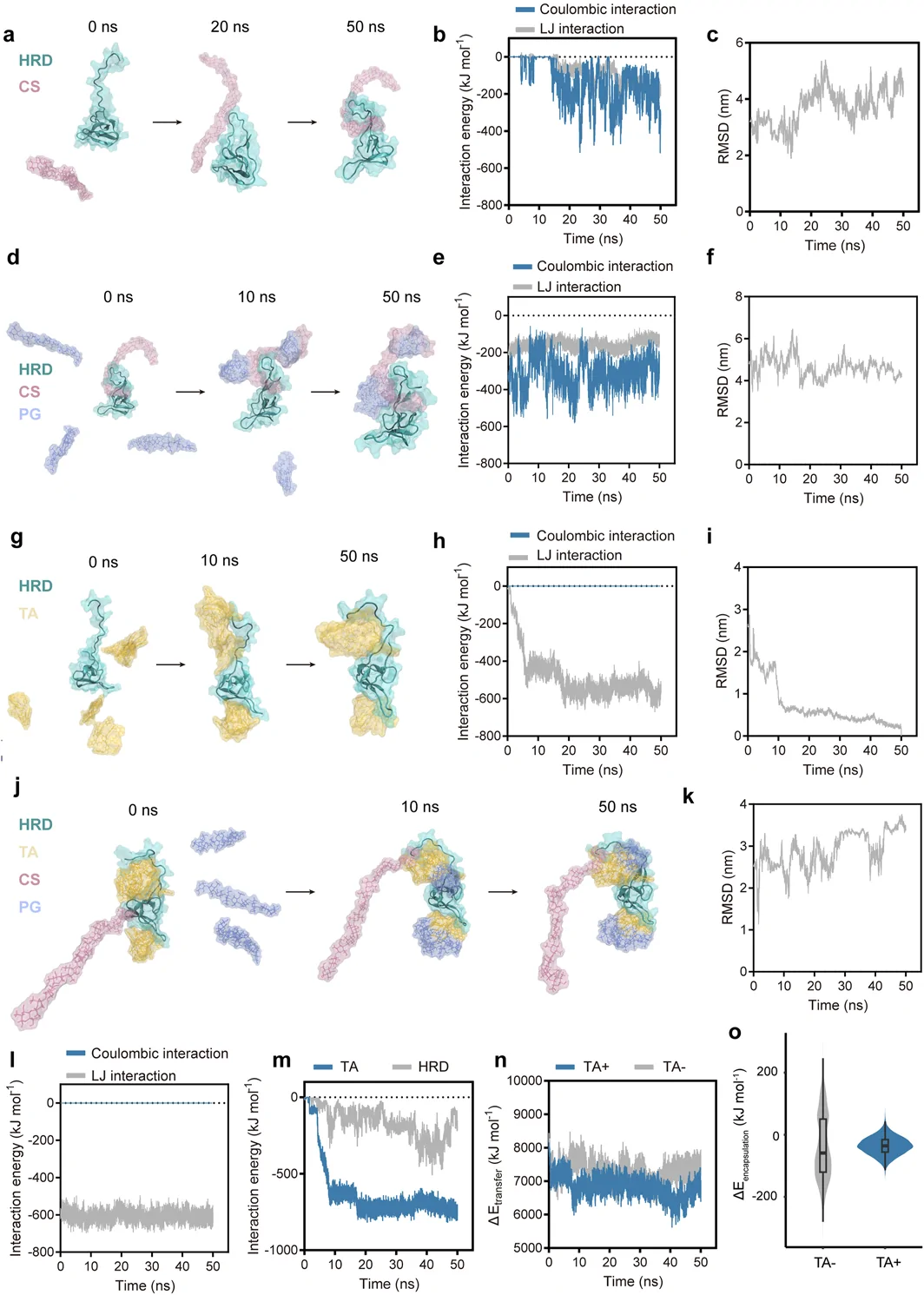
Molecular dynamics simulation depicts TA mediated HRD encapsulation mechanism. (a) Representative simulation snapshots of HRD‐CS interaction in the absence of TA; (b) Time evolution of Coulombic and Lennard–Jones (LJ) interaction energies, and (c) root mean square deviation (RMSD) profiles showing dynamic and fluctuating binding behavior; (d) Sequential introduction of PG during assembly alters HRD‐CS interactions, as reflected by representative configurations, (e) interaction energy profiles, and (f) increased RMSD fluctuations; (g) Representative simulation snapshots of HRD‐TA interaction, where TA rapidly adsorbs onto HRD and reaches a stable configuration; (h) The interaction is dominated by LJ interactions and (i) exhibits more stable structure as evidenced by the reduced RMSD variation; (j) Sequential introduction of CS and PG to TA‐HRD complex delineating the encapsulation of the protein complex within the polysaccharide network, and (k) corresponding RMSD evolution, as well as (l) the interaction energy profiles are also evaluated; (m) Decomposition of interaction energies between HRD and surrounding components; (n) Calculated energy transfer with or without the presence of TA during simulation; (o) Distribution of encapsulation energy changes before and after NPs formation with or without the introduction of TA.

We next evaluated the interaction between TA and HRD, where TA rapidly adsorbed onto HRD and reached a stable configuration within 10 ns (Figure 4g). In contrast to CS‐mediated binding, the TA‐HRD association was exclusively governed by van der Waals (vdW) interactions, as reflected by the Lennard–Jones (LJ) potential (Figure 4h). The conformation remained stable during the following simulation, as evidenced by minimal RMSD variation (Figure 4i). Importantly, when CS and PG were further introduced, the TA‐HRD complex remained intact and gradually embedded within the polysaccharide network (Figure 4j). Despite the overall RMSD of the TA‐HRD complex showing oscillations (Figure 4k), the vdW energy remained stable during the whole simulation (Figure 4l). Notably, CS and PG primarily interacted with the TA layer rather than the HRD surface (Figure 4m), supporting a model in which TA pre‐coating can protect HRD from electrostatic perturbation during the NPs assembly.

Consistent with this mechanism, the calculated energy transfer (Δ*E*_transfer_ = *E*_carrier‐HRD_ − E_water‐HRD_) decreased markedly in the TA‐coating setting (Figure 4n), indicating a stronger encapsulation within the NPs. Furthermore, the fluctuation in carrier‐HRD interaction energy before and after NPs formation was substantially smaller for the TA + setting (Figure 4o), confirming that TA minimizes the structural disturbance during the encapsulation process. In conclusion, these results reveal that TA reprograms the encapsulation mechanism from electrostatic adsorption to robust vdW‐driven stabilization, thereby “lock‐in” the protein within the polysaccharide matrix. Such polyphenol‐polysaccharide intermolecular driven stabilization is also observed in nature derived biomaterial platforms, including 3D‐printed okra‐based hemostatic sponges and magnetic pollen micromotors for oral disease treatment [48, 49].

To experimentally examine whether electrostatic interactions were essential for TAPE‐mediated protein encapsulation, we further challenged the system under different ionic‐strength conditions. If encapsulation were predominantly driven by electrostatic attraction, increasing ionic strength would be expected to weaken protein‐carrier association through charge screening and consequently reduce the encapsulation efficiency. We therefore compared the EE of TAPE in pure water, 1× PBS, 10× PBS, and 10% NaCl solution. Notably, the EE remained largely unchanged despite the substantial increase in ionic strength (Supporting Information S1: Figure S5), indicating that protein retention in TAPE is highly resistant to electrostatic screening. This experimental observation is consistent with the MD simulations and further supports a predominantly non‐electrostatic TA‐mediated stabilization mechanism.

### 2.5 Safety Evaluation

Prior to the following biological evaluation, we first assessed their safety profile both in vitro and in vivo. In vitro biocompatibility assessments were conducted by co‐incubating each delivery system with murine endothelial cells (bEND.3) and human monocytic cells (THP‐1) at a range of concentrations. Cell viability was quantitatively determined using the CCK‐8 assay, demonstrating that none of the four polysaccharide carriers induced significant cytotoxicity at concentrations ranging from 10 to 100 μg·mL^−1^ (Figure 5a,b). Subsequently, in vivo safety was evaluated in mice following intravenous (i.v.) administration of different formulations at a dosage of 2 mg·kg^−1^. Biochemical analyses of plasma markers (ALT, AST, and creatinine) at 24 h post‐injection revealed no statistically significant alterations in hepatic or renal function parameters compared to the control group (Figure 5c), partly indicating that the evaluated TAPE systems exhibit favorable safety profiles, supporting their potential as carriers for therapeutic proteins for the sequential experiments.

**FIGURE 5:**
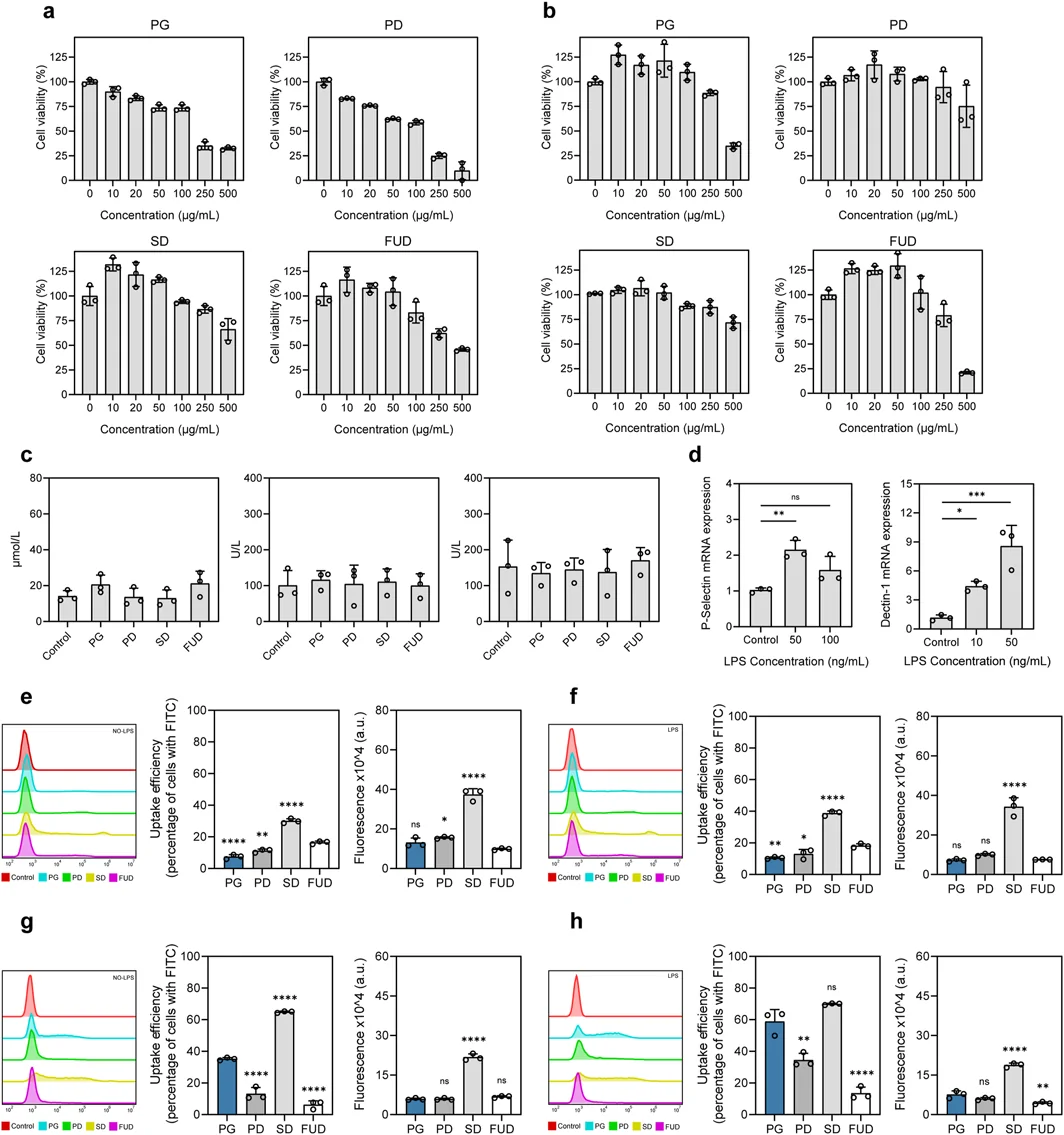
The safety evaluation and in vitro targeted validation of the TAPE system. (a) bEND.3 cells viability under varying concentrations of different TAPE systems. (b) THP‐1 cells viability under varying concentrations of different TAPE systems. (c) The levels of creatinine, ALT and AST in the plasma of mice after tail vein injection of the TAPE system. (d) In the different LPS concentrations, the expression of P‐selectin in the bEND.3 and the expression of Dectin‐1 in THP‐1. (e) The targeting ability of bEND.3 to different TAPE systems without LPS stimulation. (f) The targeting ability of bEND.3 to different TAPE systems with LPS stimulation. (g) The targeting ability of THP‐1 to different TAPE systems without LPS stimulation. (h) The targeting ability of THP‐1 to different TAPE systems with LPS stimulation.

### 2.6 TAPE With Tailored APs Enable Programmable Targeting of Cell Subsets In Vitro

One major advantage of the current TAPE system is the programmable targeting capability through tailoring the selection of APs. The targeting mechanism relies on intrinsic lectin‐glycan recognition, where disease‐associated lectins selectively bind to certain polysaccharide motifs [50]. Within our tested APs, sulfated glycans can be used to target damaged endothelial cells through the recognition by P‐selectin, whereas β‐glucan is a known precise ligand for Dectin‐1 on phagocytic immune cells. Therefore, to test whether tailoring the AP component within TAPE could direct protein carriers toward specific cell types, we examined cellular interaction in bEND.3 and THP‐1 cells.

To validate this targeting rationale, we first examined the expression dynamics of these lectins under pathological conditions. In bEND.3 endothelial cells, qPCR analysis revealed a significant upregulation of P‐selectin upon LPS stimulation, confirming injury‐associated P‐selectin activation. Similarly, Dectin‐1 expression was markedly elevated in LPS‐stimulated THP‐1 cells, consistent with macrophage inflammatory activation (Figure 5d).

Supported by the lectin expression pattern, FITC‐labeled CS was used to construct TAPE formulations, enabling quantitative analysis of cellular uptake by flow cytometry (FACS). In endothelial cells (bEND.3), sulfated AP‐based systems (SD‐TAPE and FUD‐TAPE) exhibited the highest uptake, followed by PG‐ and PD‐based formulations (Figure 5e). Moreover, upon LPS stimulation (50 ng·mL^‐1^), which markedly upregulated P‐selectin expression, the uptake of SD‐TAPE and FUD‐TAPE further increased (Figure 5f), consistent with the well‐established affinity between sulfated APs and P‐selectin.

In monocytic THP‐1 cells, SD‐TAPE also showed high uptake with or without the LPS stimulation (10 ng·mL^‐1^). However, in contrast to the limited uptake in bEND.3 cells, PG‐TAPE displayed a sharp increase in cellular interaction in THP‐1 cells, and is further increased after LPS activation, aligning with the elevated expression of Dectin‐1 (Figure 5g,h). Moreover, compared to bEND.3 cells, FUD‐TAPE showed diminished uptake in THP‐1, while PD‐TAPE consistently exhibited the lowest cellular interaction across all conditions.

To quantitatively assess targeting selectivity in different cells, we defined the endothelial targeting index (ETI = positive percentage or fluorescence intensity of bEND.3^+^/THP‐1^+^ ratio) and macrophage targeting index (MTI = 1/ETI). Despite SD‐TAPE showing high uptake in both cell types, FUD‐TAPE achieved the highest ETI, confirming its endothelial preference, whereas PG‐TAPE displayed the highest MTI, consistent with macrophage tropism (Figure 6a). Collectively, we showed that by tailoring the AP component, the systems demonstrated tunable targeting, enabling preferential delivery to lesional macrophages, endothelial cells, or reduced cellular interaction, which are potentially useful for long circulation.

**FIGURE 6:**
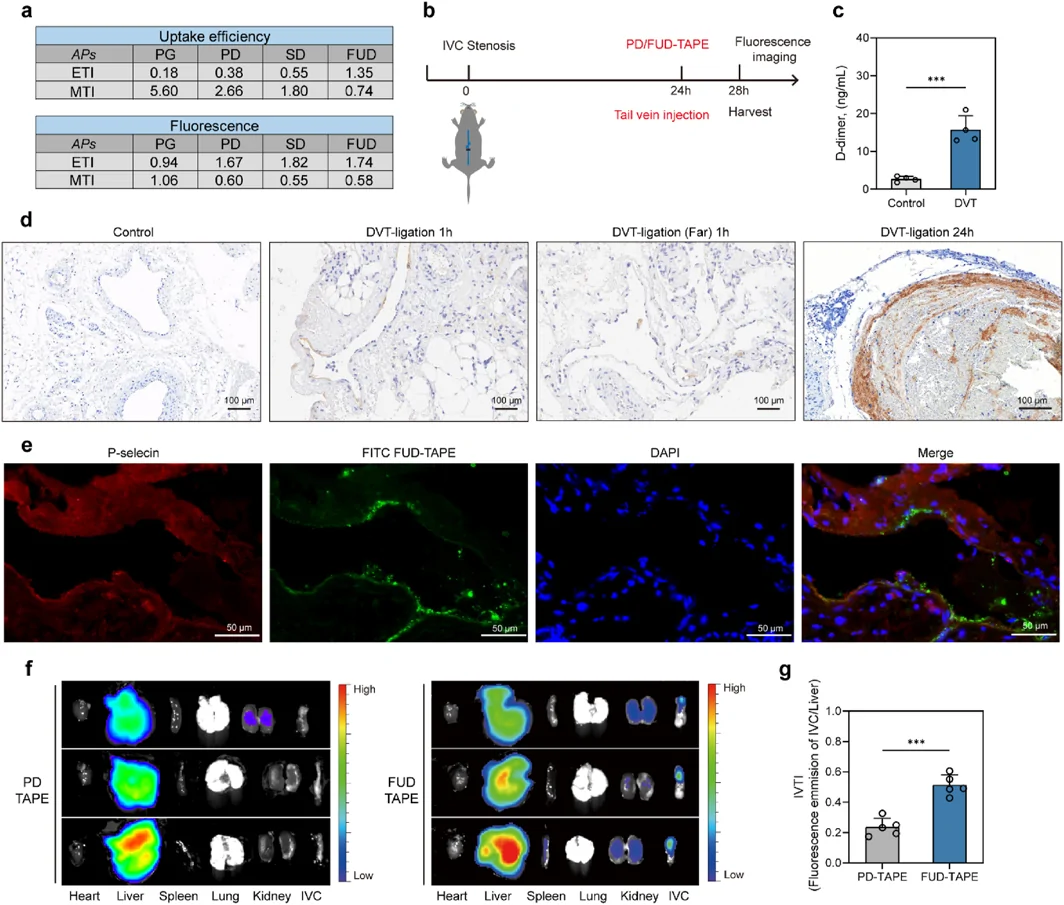
The programmable targeting capability of the TAPE system and the enrichment validation of FUD‐TAPE in the DVT model of endothelial damage. (a) Assessment of target selectivity in different cell types. (b) Experimental design. (c) D‐dimer levels in mouse tail vein blood samples at 24 h. Data are presented as mean ± SD, *n* = 4; ***p < 0.001. (d) Representative immunohistochemical staining (P‐selectin) results of the inferior vena cava ligated and unligated sites at different time points; Scale bar: 100 μm. (e) Representative immunofluorescence staining (P‐selectin, FITC, DAPI) results of the ligated end of the inferior vena cava; Scale bar: 50 μm. (f) Representative IVIS imaging of ICG‐PD and ICG‐FUD in major organs, including (from left to right) heart, liver, spleen, lung, kidney and injured IVC. (g) Fluorescence emission of IVC/liver. Data are presented as mean ± SD, *n* = 5; ***p < 0.001.

### 2.7 FUD‐TAPE Exhibits Enhanced Accumulation at Injured Endothelium In Vivo

Having established the programmable targeting capacity of the TAPE system in vitro, we next sought to evaluate whether such selectivity could be recapitulated in vivo. Among the tested formulations, FUD‐TAPE exhibited tropism toward injured endothelium, a feature with high clinical relevance which can be observed in a broad spectrum of vascular disorders [51, 52]. Therefore, we employed a murine deep vein thrombosis (DVT) model through a surgical blood flow obstruction in the inferior vena cava (IVC), characterized by acute endothelial injury [53, 54], to test the targeting capability of FUD‐TAPE in a pathophysiological context (Figure 6b). The successful induction of the DVT model was confirmed by the elevated D‐dimer value at 24 h post the surgical procedure (2.69 ± 0.63 v.s. 15.71 ± 3.21, Control v.s. DVT, Figure 6c). We next examined P‐selectin expression in the different vasculature microenvironments. P‐selectin was markedly upregulated in the vasculature at 1 h post‐DVT induction, whereas its expression remained minimal in healthy IVC tissues. Notably, this elevation was majorly localized to thrombus‐associated regions and was not observed in adjacent normal vessels. The enhanced P‐selectin expression persisted for at least 24 h, indicating sustained endothelial activation at the injury site (Figure 6d). We next examined whether FUD‐TAPE can recognize and bind P‐selectin rich regions. Confocal microscopy revealed substantial colocalization between fluorescence labeled FUD‐TAPE (green) and P‐selectin (red) signals, confirming that the endothelial targeting of FUD‐TAPE is mediated by P‐selectin (Figure 6e).

With the validated overexpression of P‐selectin in the DVT model, we next evaluated the in vivo biodistribution of FUD‐TAPE. For comparison, PD‐TAPE, which showed limited cellular interaction in vitro, was used as a negative control. Mice were intravenously injected with either indocyanine green (ICG) labeled FUD‐TAPE or PD‐TAPE at 24 h following DVT induction, and were sacrificed at 4 h post‐injection. Major organs, including the heart, liver, spleen, lungs, kidneys, and the injured IVC, were collected for ex vivo fluorescence imaging. Pronounced accumulation of FUD‐TAPE was observed at the injured IVC, in contrast to the limited signal from PD‐TAPE (Figure 6f). To quantify targeting selectivity, we defined an injured vasculature targeting index (IVTI), calculated as the ratio of fluorescence intensity between the injured IVC and the liver (IVTI = Fluorescence emission of IVC/Liver). The PD‐TAPE group exhibited an IVTI of 0.24 ± 0.05, which was significantly lower than that of FUD‐TAPE (0.51 ± 0.06, Figure 6g). These results confirm that the FUD‐TAPE formulation confers enhanced specificity toward sites of vascular injury, consistent with its affinity for P‐selectin expressing endothelium.

### 2.8 FUD‐TAPE‐HRD Achieved an Enhanced Anticoagulant Therapy

Lastly, we sought to demonstrate whether the TAPE system can translate into functional therapeutic outcomes in vivo. Guided by the previously established affinity of FUD‐TAPE toward P‐selectin overexpressed endothelium, we designed to encapsulate the clinically validated thrombin inhibitor HRD within the FUD‐TAPE for enhanced anticoagulant delivery. To evaluate whether the FUD‐TAPE system could sustain the anticoagulant activity of HRD, we first assessed its coagulation inhibition in vitro using a needle‐prick assay. Among the tested groups (saline, free HRD, FUD‐TAPE, and FUD‐TAPE‐HRD, corresponding to 0.01 mg/mL^−1^ HRD), free HRD showed the strongest anticoagulant effect. Blank FUD‐TAPE also exhibited mild inhibition of coagulation, consistent with previous reports suggesting the intrinsic anticoagulant activity of FUD [55, 56, 57]. Notably, the FUD‐TAPE‐HRD formulation displayed enhanced inhibition compared to blank FUD‐TAPE and is non‐inferior to free HRD at the same concentration, confirming that the nanocarrier preserved HRD bioactivity (Figure 7a).

**FIGURE 7:**
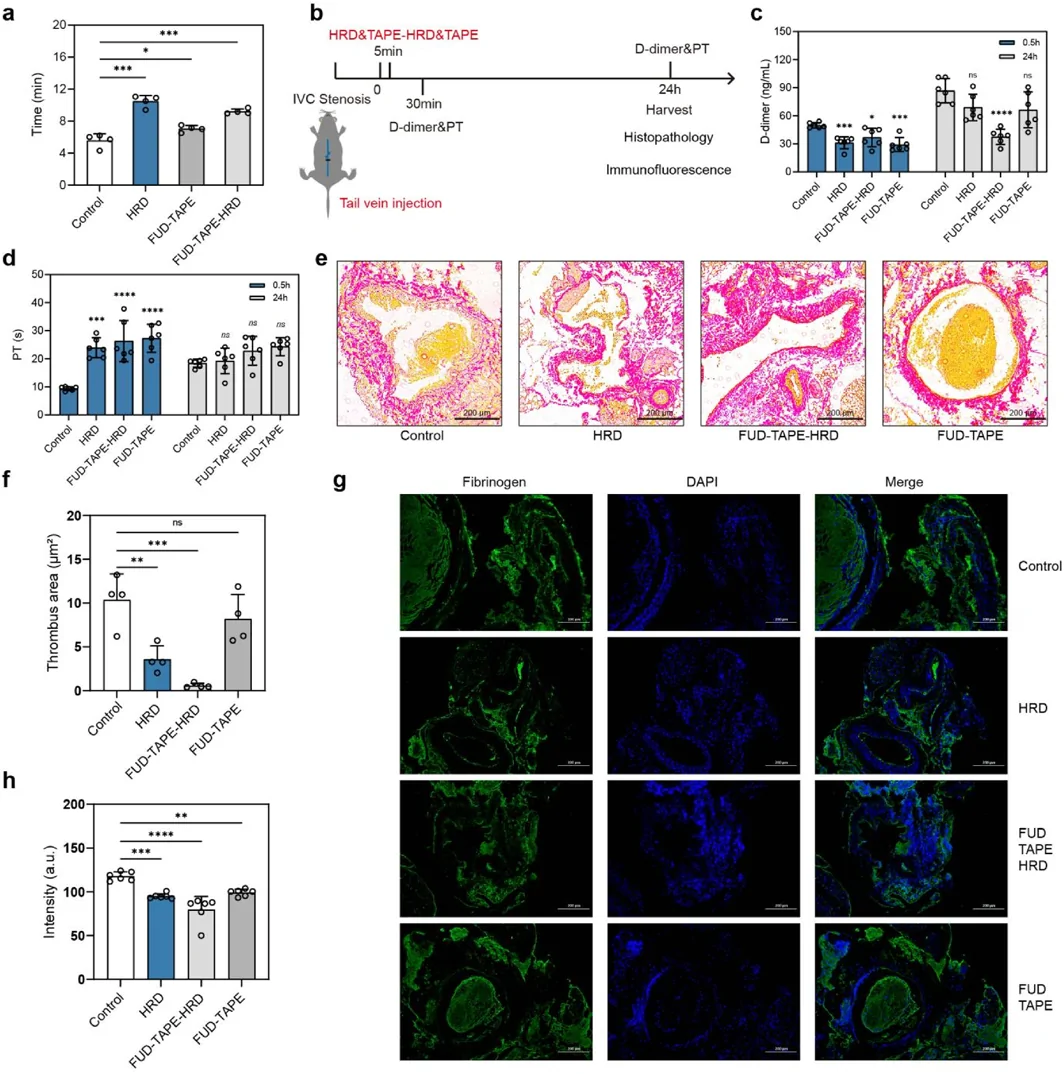
Validation of the FUD‐TAPE‐HRD system for enhanced anticoagulant therapy in DVT mice. (a) The anticoagulant effect of different materials was detected using acupuncture method. Data are presented as mean ± SD, *n* = 4; *p < 0.05; ***p < 0.001. (b) Experimental design. (c) D‐dimer levels in mouse tail vein blood samples at 0.5 and 24 h. Data are presented as mean ± SD, *n* = 6; *p < 0.05; ***p < 0.001; ****p < 0.0001. (d) PT values in mouse tail vein blood samples at 0.5 and 24 h. Data are presented as mean ± SD, *n* = 6; ***p < 0.001; ****p < 0.0001. (e) Representative picro‐sirius red staining results of the inferior vena cava ligated sites; Scale bar: 200 μm. (f) Quantitative analysis of picro‐sirius red staining results of the inferior vena cava ligated sites. Data are presented as mean ± SD, *n* = 4; **p < 0.01; ***p < 0.001. (g) Representative immunofluorescence staining (Fibrinogen, DAPI) results of fibrin in the vasculature; Scale bar: 200 μm. (h) Quantitative analysis of immunofluorescence staining (Fibrinogen, DAPI) results of fibrin in the vasculature. Data are presented as mean ± SD, *n* = 4; **p < 0.01; ***p < 0.001; ****p < 0.0001.

We next examined whether these effects can be translated in vivo in a murine DVT model. The experimental timeline was designed to assess both short‐ and long‐term coagulation responses (Figure 7b). At 5 min post‐DVT induction, mice received intravenous administration of saline, free HRD, FUD‐TAPE, or FUD‐TAPE‐HRD (equivalent to 2 mg·kg^−1^ HRD). Prothrombin time (PT) and plasma D‐dimer levels were measured at 30 min and 24 h post‐injection.

30 min post administration, all formulations significantly prolonged PT and reduced D‐dimer levels compared with the untreated DVT group, aligning with the in vitro findings and indicating short‐term anticoagulant efficacy from HRD and FUD‐containing formulations. However, after 24 h, only the FUD‐TAPE‐HRD group maintained a marked reduction in D‐dimer levels, whereas PT values remained relatively high across all groups (Figure 7c,d). Histological analysis was performed using picro‐sirius red staining to evaluate thrombus formation. Extensive embolization and endothelial disruption were evident in the model group. In contrast, mice receiving FUD‐TAPE‐HRD showed the lowest degree of thrombus deposition and minimal endothelial damage, with the clot area drastically diminished relative to saline controls (Figure 7e,f).

To further verify the anticoagulant effect, immunofluorescence (IF) staining was conducted to assess fibrin accumulation within the vasculature. Consistent with the H&E findings, the FUD‐TAPE‐HRD group exhibited the weakest fibrin (green) signal among all groups, confirming the potent anticoagulant activity of HRD‐TPP in vivo (Figure 7g,h).

Collectively, these results suggest that the FUD‐TAPE‐HRD formulation not only achieves effective short‐term coagulation control but also sustains prolonged antithrombotic protection through targeted delivery and stable protein encapsulation.

## 3 Conclusion

Protein therapeutics have long been limited by instability and lack of targeting capability, and there is a longstanding challenge in achieving both stable encapsulation and selective delivery of proteins. In this study, we propose a modular and programmable polysaccharide‐based platform, termed TAPE, to enable efficient protein encapsulation, broad applicability across diverse proteins, and tunable targeting capability. Compared with conventional electrostatic complexation approaches that rely on cationic vehicles, such as CS, to bind negatively charged proteins, TAPE achieved markedly higher encapsulation efficiency with robust reproducibility across diverse proteins, independent of their isoelectric points or formulation conditions. In addition, by tailoring the selection of interchangeable AP components, TAPE enables programmable targeting, allowing its delivery profile to be customized for distinct therapeutic needs or protein cargos.

## 4 Materials and Methods

Materials: Hirudin (10 mg/mL^−1^, purity ≥ 95% by electrophoresis, recombinant, concentrated solution), purchased from Shanghai Yuanye Bio‐Technology Co. Ltd; Insulin, purchased from Acmec; Albumin, purchased from Yeasen; Chitosan, β‐glucan (Barley; Low Viscosity), purchased from Megazyme; Tannic acid (TA), dextran sulfate sodium (SD), purchased from Acmec; Dextran D20, purchased from Meilunbio; Fucoidan (purity ≥ 95%), purchased from Aladdin; Polyphosphate, trimethylamine, formamide, DMSO, FITC, carbonate buffer (pH 9), purchased from Solarbio.

Preparation of NPs and TAPEs: Chitosan as the cationic component is immobilized, and further co‐assembled with different types of APs in aqueous solution, including PD, SD, PG and FUD. The chitosan concentration (1 mg/mL^−1^ in 1% acetic acid solution, pH 5) is kept constant, different relative weight ratios of APs to CS (AP/CS) are adopted, and the solutions are mixed at equal volume ratios, followed by ultrasonication to form NPs. For TAPEs preparation, first prepare TA aqueous solution at corresponding concentration according to the molar ratio of protein to TA, then rapidly mix protein and TA by vortexing, sequentially add CS and APs solutions following the above NPs preparation procedure, and homogenize by ultrasonication to obtain TAPEs.

Preparation of phosphorylated β‐glucan/Dextran: β‐glucan/Dextran (1 g) and DMSO (20 mL) were stirred at 70°C until fully dissolved. Subsequently, 4 mL of triethylamine and 1 g of polyphosphoric acid were added. The mixture was then stirred at 70°C for 48 h. Next, 50 mL of ethanol was added, and the solution was left to precipitate overnight at 4°C. The precipitate was then centrifuged, redissolved in 10 mL of water, and freeze‐dried after dialysis.

Preparation of FITC‐Protein: The FITC‐protein conjugates were prepared by initially dissolving protein (4 mg/mL^−1^, respectively BSA, Insulin, OVA and Hirudin) in pH 9 carbonate buffer and FITC (4 mg/mL^−1^) in DMSO, followed by the controlled addition of the FITC solution to the protein solution at a 50:1 mass ratio under continuous stirring. The reaction mixture was then incubated overnight in complete darkness, after which the product was subjected to 48‐h dialysis and lyophilization.

FITC labeling of CS: 100 mL of dehydrated methanol followed by 50 mL of FITC in methanol (2 mg/mL^−1^) was added dropwise to 100 mL of chitosan dissolved in 0.1 m acetic acid (1%) in the dark at room temperature. The reaction was kept for 3 h under magnetic stirring so that the amino group on chitosan reacted with the isothiocyanate group on FITC to yield a thiourea linkage. The labeled polymer was then precipitated in 0.2 m NaOH, pelleted at 40,000 g for 10 min and washed several times with methanol:water (70:30, v/v) until no fluorescence was detected in the supernatant (*λ*exc = 490 nm, *λ*emi = 520 nm).

Preparation of ICG‐Chitosan: The ICG‐labeled chitosan was prepared by adjusting the pH of a chitosan solution (1 mg/mL^−1^) to 8–9, followed by the addition of ICG‐NHS (1622335‐40‐3) at a 1:20 mass ratio under continuous stirring overnight in darkness. The reaction mixture was then dialyzed for 48 h in the dark using a dialysis bag and subsequently lyophilized. The obtained ICG‐chitosan was utilized to synthesize the delivery systems for PD and FUD.

Diameter and zeta potential measurements: The diameter and zeta potential of NPs were evaluated using a ZetaSizer NanoSeries (Zetasizer Nano ZS‐90, Malvern Instruments). The relative weight ratios for the concentrations of APs and CS (AP/CS) were set to 0.25, 0.5, 1, 2.5 and 5 by mixing 1:1 volumetric ratios while maintaining a constant concentration of CS. The samples were prepared by mixing 100 μL of chitosan (1 or 0.2 mg/mL^−1^ in 1% acetate buffer, pH 5) with 100 μL of PG, PD, SD, or FUD (all dissolved in ultrapure water) at room temperature, followed by dilution to 800 μL with ultrapure water. Particle size and Zeta potential were subsequently measured. The four polysaccharide delivery systems loaded with hirudin (0.4 mg/mL^−1^) and the particle sizes were measured as well.

Transmission electron microscopy (TEM): Following synthesis, the nanoparticle suspension was diluted and deposited onto carbon‐coated copper grids under contaminant‐free conditions, with grids handled exclusively at the edges using clean tweezers to prevent film damage. After immersion for homogeneous nanoparticle adhesion, excess liquid was removed with lint‐free filter paper and the samples pre‐dried under an infrared lamp before TEM imaging.

Dialysis and lyophilization: Following 48‐h dialysis against ultrapure water at room temperature using 3.5 kDa regenerated cellulose dialysis bags (FDM503‐5m, Beyotime/Biyuntian), the samples were collected and lyophilized. Freeze‐drying protectants including glycine, mannitol, and maltose (1%, 2% or 3%) were utilized. The particle size distribution before and after lyophilization was evaluated.

Measurement of protein encapsulation efficiency: For APs, the protein encapsulation efficiency was determined by quantifying the unencapsulated hirudin in the supernatant using a BCA assay kit (Sigma). Chitosan solutions (1, 2, or 5 mg/mL^−1^ in 1% acetate buffer, pH 5) were mixed with hirudin (0.1, 0.2, or 0.4 mg/mL^−1^) and polysaccharides (PG/SD/PD/FUD at 0.5, 1, 5, or 10 mg/mL^−1^ in water). After thorough vortexing and centrifugation at 14,000 rpm for 8 min, the supernatant was collected for BCA protein quantification, with encapsulation efficiency calculated based on the difference between initial and residual hirudin concentrations. For the TAPE system, the protein encapsulation efficiency was assessed by measuring the fluorescence intensity of FITC‐labeled proteins in solution. We used BSA as the model protein for the subsequent experiments. FITC‐BSA/TA molecular ratios (1:10/20/40/80/120) and various APs were systematically evaluated. Following thorough mixing, samples were centrifuged at 14000 rpm for 8 min, and the supernatant was collected. Fluorescence intensity of FITC in the supernatant was quantified using a multi‐label detection system (Envision, PE, Xiamen University Biological Instrument Shared Platform) with excitation at 488 nm and emission at 524 nm.

Measurement of protein loading capacity: The protein loading capacity (LC) of different TAPEs was quantitatively calculated based on the encapsulation efficiency data measured in the aforementioned section. Briefly, the total mass of fully encapsulated protein was first derived by multiplying the total initial input mass of protein by the corresponding experimentally determined encapsulation efficiency of each formulation, which excluded the amount of free protein leaked during the preparation process. Subsequently, the total mass of the TAPE system was calibrated according to the preset formulation ratio of each group, which was defined as the sum of the actual weighed mass of three core components (TA, Chitosan and APs), without counting the mass of unencapsulated free protein in the supernatant. Finally, the loading capacity of TAPEs was calculated as the ratio of the total mass of fully encapsulated protein to the calibrated total mass of the corresponding TAPE system.

Protein release from different TAPE systems in vitro: The protein release from different TAPE systems was assessed using FITC‐labeled BSA as a model protein. The release of FITC‐BSA was measured within 24 h at 37°C in phosphate‐buffer solution (PBS) containing 5% FBS (simulating the physiological environment) and in a low‐pH environment (pH 4.0, PBS containing 5% FBS) by collecting samples at different time points. After centrifugation, the supernatant was collected and the protein content was quantitatively analyzed to reflect the release of particulate. Finally, the data were fitted using the formula *Y* = *B*_max_ × *X*/(Kd + *X*) and the release curve was plotted.

Extreme condition release of proteins in different TAPE systems: PBS solution containing 5% fetal bovine serum (FBS) was prepared as the background control. For the negative control, 2 μL of FITC‐BSA was added to 1 mL of 5% FBS‐PBS. For the positive control, 2 μL of FITC‐BSA was mixed with 1 mL of TA (2 mg/mL^−1^). Four polysaccharide‐based delivery systems were synthesized by combining 2 μL of FITC‐BSA with polysaccharides. After synthesis, the volume was adjusted to 1 mL with 5% FBS‐PBS. A 100 μL aliquot was taken for fluorescence intensity measurement, and centrifugation. The supernatant was removed, and the particle was resuspended in 1 mL of 5% FBS‐PBS via sonication. This measure‐wash‐resuspend cycle was repeated five times.

Molecular dynamics simulation: All molecular dynamics (MD) simulations were performed using GROMACS 2021.5 with the Amber99SB force field. The construction of polymeric molecules was conducted in ChemDraw (CambridgeSoft, U.S.). All molecular structures were protonated at pH 5 using OpenBabel (v2.3.1), and force fields generated using the Sobtop (dev3.1). The pdb file of HRD was obtained from the AlphaFold Protein Structure Database, and the force fields were generated using the gmx pdb2gmx tool. The initial simulation box size was 8 nm in each dimension (*x*, *y*, and *z*). The simulation box includes HRD and pairing molecules solvated in OPC3 water molecules. The system was first subjected to energy minimization using the L‐BFGS algorithm until the maximum force was below 500 kJ·mol^−1^·nm^−1^. Production simulations were conducted in the NPT ensemble using a time step of 2 fs for 50 ns (25,000,000 steps). The system temperature was maintained at 298.15 K using the V‐rescale thermostat. Long‐range electrostatics were handled using the Particle Mesh Ewald (PME) method with a cutoff of 1.0 nm. van der Waals interactions were truncated at 1.0 nm, and dispersion corrections for energy and pressure were applied. All bonds involving hydrogen atoms were constrained using the LINCS algorithm. Interaction energy and root mean square deviation (RMSD) were determined using the gmx energy and gmx rms tools, respectively, that are provided with the GROMACS package. Visual Molecular Dynamics (VMD) program was used to visualize and render the figures. To quantify structural perturbation during NP assembly, we calculated the change in HRD‐carrier interaction energy before and after encapsulation. For each system, the total interaction energy between HRD and its immediate binding partner (HRD‐TA in TA + systems and HRD‐CS in TA‐systems) was obtained by summing the Coulombic and van der Waals (vdW) contributions. The mean interaction energy was first computed from the final 1 ns of the pre‐assembly trajectory, representing the stabilized protein‐ligand complex prior to NPs formation. Following the introduction of PG to initiate NPs assembly, interaction energies were extracted at every frame during the first 10 ns of the post‐assembly trajectory. The difference between each post‐assembly time point and the pre‐assembly baseline was calculated to yield the interaction energy shift (Δ*E*encapsulation). The resulting distributions were visualized as violin plots to compare structural perturbation across formulations.

Animals: Male C57BL/6J mice (Vitolab Laboratory Animal Technology Co. Ltd) aged 9–10 weeks were housed in a SPF facility at the Animal Observation Laboratory of Xiamen University School of Pharmacy. The environmental conditions were controlled and maintained at 25 ± 2°C with 50%–75% relative humidity. The mice had free access to water and standard chow and were kept on a 12‐h light/dark cycle. All animal husbandry and experimental procedures were approved by the Animal Ethics Committee of Xiamen University (Ethical Approval No.: XMULAC20240276) and complied with the Animal Research: Reporting of In Vivo Experiments (ARRIVE) guidelines.

Biosafety evaluation: In vitro biocompatibility of each delivery system was assessed by co‐incubating bEND.3 vascular endothelial cells and THP‐1 monocytic cells (differentiated into macrophages using 300 ng·mL^−1^ phorbol PMA) with synthesized particles across a concentration gradient for 24 h at 37°C, followed by CCK‐8 assay (Gibco) incubation and absorbance measurement at 450 nm to quantitatively determine cell viability. In vivo biocompatibility of each delivery system was evaluated by administering APs to mice via tail vein at 2 mg·kg^−1^ (with saline as control), after which plasma and liver samples were collected at 24 h and analyzed for ALT, AST and creatinine levels using an automated biochemical analyzer (iMagic‐M7, icubio) to assess potential acute hepatorenal injury.

RT‐qPCR: After cell cultivation, different concentrations (0–500 ng·mL^−1^) of LPS were, respectively, administered to induce the cells. Total RNA was collected and the expression of Dectin‐1 in THP‐1 and the expression of P‐selectin in BEND.3 were detected under different LPS concentrations. Total RNA was isolated using a commercial kit (Vazyme), followed by reverse transcription into cDNA with the 2× Hifair II SuperMix plus cDNA synthesis kit (Yeasen), and PCR amplification was performed on a QuantStudio 6 real‐time fluorescence quantitative PCR system (ABI). The relative RNA expression levels were quantified using the 2^^−ΔΔCt^ method, with primer sequences detailed in Supporting Information S1: Table S2.

In vitro targeted experiment: The cells were divided into the LPS group and the non‐LPS group. THP‐1 was inoculated and incubated with 300 ng·mL^−1^ PMA for 24 h to differentiate into macrophages. After 24 h, the fresh culture medium was replaced and BEND.3 were inoculated without any treatment. 50 ng·mL^−1^ LPS was added to the LPS group, while the NO‐LPS group was not treated. After another 24 h of incubation, all wells were replaced with culture medium and four polysaccharide delivery systems containing FITC‐chitosan (10 μg·mL^−1^) were added. After incubation for 4 h, the cells were washed 3–5 times with preheated PBS at 37°C to remove unbound particles, and then the cells were harvested into a suspension for flow cytometry analysis to quantify fluorescence intensity and polysaccharide uptake. Flow cytometric analysis was performed on a CytoFLEX LX (Beckman). Statistical comparisons were made between the LPS/NO‐LPS group and the polysaccharide types to evaluate the cell response.

DVT model: Under continuous anesthesia maintained via a 5% isoflurane mask, the mice underwent standardized surgical procedures commencing with abdominal hair removal followed by thorough disinfection using povidone‐iodine solution, after which a midline abdominal incision was meticulously performed to expose the visceral cavity. The inferior vena cava and abdominal aorta were subsequently bluntly dissected. Precise separation was achieved using a 30G needle as a surgical spacer. Prior to ligation of both the spacer and the inferior vena cava with 6–0 sutures, this technique was deliberately employed to establish approximately 90% vascular occlusion upon needle removal. The surgical site was then closed with sutures under aseptic conditions, with postoperative monitoring conducted until complete restoration of locomotor function was confirmed.

Diabetic model: Type I diabetes was induced by a single intraperitoneal (i.p.) injection of streptozotocin (STZ; Sigma‐Aldrich, St. Louis, MO, USA) at a dose of 200 mg·kg^−1^. STZ was dissolved in 50 mM sodium citrate buffer (pH 4.5) immediately before administration. Blood glucose levels were measured 72 h after STZ injection, and mice with blood glucose concentrations > 16.7 mmol·L^−1^ were considered diabetic and included in subsequent experiments.

Blood glucose measurement: Diabetic mice were randomly assigned to four groups: blank FUD‐TAPE, free insulin (INS), insulin‐loaded FUD‐TAPE (FUD‐TAPE‐INS), and insulin released from FUD‐TAPE (FUD‐TAPE‐INS‐RE). To prepare the released‐insulin fraction, FUD‐TAPE‐INS was dispersed in 0.5 mL PBS containing 10% bovine serum albumin (BSA), incubated at 37°C with sonication for 5 min, and subsequently centrifuged. The resulting supernatant containing released insulin was collected for administration.

Before treatment, the mice were fasted for 12 h with free access to water. The corresponding formulations were administered by intraperitoneal (i.p.) injection. For all insulin‐containing groups, the insulin concentration was adjusted to 0.133 mg/mL^−1^, and the administration volume was 3 μL·g^−1^ body weight. Blood samples were collected from the tail vein immediately before administration (0 h) and at 0.5, 1, and 1.5 h after treatment. Blood glucose concentrations were measured using a Sinocare portable glucometer.

Long‐term release evaluation of delivery systems: In vivo assay, a single drop of orbital blood obtained from mice was carefully placed onto a glass slide and systematically assigned to 4 groups: the control group (saline, 10 μL), the HRD group (HRD 0.01 mg/mL^−1^, 10 μL), FUD‐TAPE group (50% FUD‐TAPE concentration, 10 μL) and the FUD‐TAPE‐HRD group (50% FUD‐TAPE concentration, and HRD 0.01 mg/mL^−1^, 10 μL). Subsequently, the coagulation process was monitored by gently stirring each blood droplet at 5 s intervals using a sterile needle, and the precise clotting times were recorded for comprehensive comparative analysis. In the in vitro assay, DVT mice were assigned to four groups: untreated DVT group, free HRD group, FUD‐TAPE group and FUD‐TAPE‐HRD group. At 5 min post‐DVT induction, mice received intravenous administration of saline, free HRD, FUD‐TAPE, or FUD‐TAPE‐HRD (equivalent to 2.0 mg·kg^−1^ HRD). The dosing volume for the FUD‐TAPE group was meticulously matched to that of the FUD‐TAPE‐HRD group, whereby the total volume of HRD (2.0 mg·kg^−1^) combined with the vehicle in the FUD‐TAPE‐HRD group was calculated based on each animal's body weight, ensuring that the FUD‐TAPE group received an equivalent volume of vehicle solution devoid of HRD. After 30 min and 24 h, blood was collected for plasma D‐dimer (ELK1311, ELK Biotechnology) and prothrombin time (PT) measurements. The animal was sacrificed and the inferior vena cava was removed.

In vivo targeting test: The in vivo targeting test was conducted by first synthesizing ICG‐labeled chitosan‐based PD‐TAPE and FUD‐TAPE, followed by intravenous administration (2.0 mg·kg^−1^ HRD equivalent) to DVT mice (*n* = 4 per group) 24 h post‐surgery, with thrombus accumulation of the delivery systems quantified using bioluminescent small animal live imaging system (Caliper IVIS Lumina II) to comparatively evaluate fucoidan‐mediated vascular endothelial targeting efficiency.

Sirius red: The vascular samples fixed in 4% paraformaldehyde were paraffin‐embedded and sectioned. Subsequently, the samples were successively immersed in dewaxing solutions and rehydrated through a graded ethanol series. Next, the sections were stained with Sirius Red A solution at 65°C for 30 min, briefly rinsed, transferred to modified Sirius Red B solution for 2 min, and further incubated in modified Sirius Red C solution for 30 min. After thorough washing, the sections were subjected to rapid ethanol dehydration. Sirius Red Staining Kit (G1078), purchased from Servicebio.

Immunofluorescence: After fixation, the blood vessel samples (from the long‐term release experiment of the delivery system in vivo) were paraffin‐embedded and sectioned. Subsequently, the samples were successively immersed in dewaxing solutions I‐III and rehydrated through a graded ethanol series. The FITC‐labeled chitosan‐based FUD‐TAPE was synthesized and administered intravenously (2.0 mg·kg^−1^ HRD equivalent) to DVT mice (*n* = 4 per group) 24 h after surgery. Four hours after injection, the mice were sacrificed, the inferior vena cava was excised for frozen sectioning, and the sections were fixed. After antigen retrieval and natural cooling, the sections were washed in PBS on a shaking platform. The sections were blocked with 5% BSA for 0.5 h. The sections were incubated overnight at 4°C with primary antibodies (Fibrinogen, GB112088, Servicebio; P‐selectin, sc‐841, Santa Cruz), followed by incubation with secondary antibodies (Alexa Fluor 488‐labeled goat anti‐rabbit IgG, GB25303, Servicebio; CY3‐labeled goat anti‐mouse IgG, GB21301, Servicebio) for 50 min. DAPI staining was used to visualize nuclei. After the final washing, autofluorescence was quenched with reagent B for 5 min, followed by a 10 min rinsing under running water. Fluorescence microscopy was performed with excitation at 465–495 nm and emission detection at 515–555 nm for Fibrinogen detection, and excitation at 510–560 nm with emission detection at 590 nm for P‐selectin visualization.

Immunocytochemistry: The DVT model was constructed as before. At 1 h and 24 h after the model establishment, the inferior vena cava tissues were collected, and the inferior vena cava tissues of healthy mice were also collected. Detect the expression of *p*‐selectin in injured blood vessels. 1‐h samples were taken to test the ligated and unligated sections, and 24‐h samples were taken to test the ligated section. After fixation, the blood vessel samples were paraffin‐embedded and sectioned. Subsequently, the samples were successively immersed in dewaxing solutions I‐III and rehydrated through a graded ethanol series. After antigen retrieval and natural cooling, the sections were washed in PBS on a shaking platform. After removing endogenous peroxidase, 5% BSA was used to block for 0.5 h. Overnight primary antibody (P‐selectin, CD62P, AB255822, abcam) incubation at 4°C was followed by 50 min secondary antibody (S‐vision immunohistochemical poly‐detection antibody goat anti‐rabbit, G1302, Servicebio) incubation. Following DAB staining, the reaction was stopped with water, and cell nuclei were counterstained with hematoxylin.

Statistical: All data are presented as mean ± standard deviation. Significance between the two groups was analyzed using t‐tests, and multiple comparisons were performed using one‐way or two‐way analysis of variance (ANOVA). Graphs were generated using GraphPad Prism 8.0 software (GraphPad, California, USA). *p* < 0.05 was considered statistically significant.

## Author Contributions

W. W. performed the in vivo experiments, contributed to data analysis, figure preparation, and manuscript drafting. Z. P. participated in experimental studies, data analysis, figure preparation, and manuscript writing. Z. L. conceived and designed the study, performed the molecular dynamics simulations, participated in the experimental studies, data interpretation, figure preparation, and manuscript writing and revision. Y. Y., D. C., J. Z., F. Z., Y. F., S. H., R. Y., S. J., and Y. H. contributed to experimental work, data analysis, figure preparation, and/or manuscript revision. C. W. and Q. X. supervised the study, contributed to study design and data interpretation, and revised the manuscript. All authors reviewed and approved the final version of the manuscript.

## Ethics Statement

All animal husbandry and experimental procedures were approved by the Animal Ethics Committee of Xiamen University (Ethical Approval No.: XMULAC20240276) and complied with the Animal Research: Reporting of In Vivo Experiments (ARRIVE) guidelines.

## Conflicts of Interest

The authors declare no conflicts of interest.

## Supporting information


Supporting Information S1

## Data Availability

The data that support the findings of this study are available on request from the corresponding author.
