# Supplementary material for: A Modular Polysaccharide Platform With Tannic Acid‐Driven van der Waals Lock‐In for Programmable Protein Delivery
Source: Smart Med. 2026 Oct 29;5(5):e70061. doi: 10.1002/smmd.70061 (PMC13720230; doi:10.1002/smmd.70061)
Supplement: Supplementary file 1 [file SMMD-5-e70061-s001.docx]

Supplementary Materials

- **Supplementary Figure S1~5**
- **Table S1-S3**

**Supplementary Figure S1.** The interaction between TA and proteins. **(a)** The turbidity analysis of TA and hirudin at different molar ratios, **(b)** Fluorescence full-wavelength scanning (Excitation: 488 nm) after the combination of different molar ratios of TA with FITC-HRD.

**Supplementary Figure S2.** The calculated protein loading degree of different TAPE system. **(a)**The protein loading capacity (LC) of different TAPE systems at different molar ratios of FITC-BSA/TA. **(b)** The LC of insulin, ovalbumin (OVA), and hirudin by different TAPE systems.

**Supplementary Figure S3.** Protective impact of freeze-drying protectants on PD-TAPE's particle size and PDI pre- and post-lyophilization. **(a)** PDI of different TAPE systems with cryoprotectants (mannitol, glycine, maltose) at 1, 2, 3% (w/v). **(b)** Particle size of different TAPE systems with cryoprotectants (mannitol, glycine, maltose) at 1, 2, 3% (w/v).

**Supplementary Figure S4.** Four delivery systems release the protein under extreme conditions, including repeated centrifugation and dissolution.

**Supplementary Figure S5.** The encapsulation efficiency of the four delivery systems in solutions of different ionic strengths

**Supplementary Taleb 1.** Orthogonal Experimental Analysis of Chitosan-Polysaccharide-Protein Concentration Effects on Delivery System Encapsulation Efficiency.

| **Grouping** | **Chitosan  (mg/mL)** | **HRD  (mg/mL)** | **polysaccharide  (mg/mL)** |
| --- | --- | --- | --- |
| **1** | 1 | 0.1 | 0.5 |
| **2** | 2 | 0.2 | 0.5 |
| **3** | 5 | 0.4 | 0.5 |
| **4** | 1 | 0.2 | 1 |
| **5** | 2 | 0.4 | 1 |
| **6** | 5 | 0.1 | 1 |
| **7** | 1 | 0.4 | 5 |
| **8** | 2 | 0.1 | 5 |
| **9** | 5 | 0.2 | 5 |
| **10** | 1 | 0.1 | 10 |
| **11** | 2 | 0.2 | 10 |
| **12** | 5 | 0.4 | 10 |

| **Grouping** | **PG** | **PD** | **SD** | **FUD** |
| --- | --- | --- | --- | --- |
|  | **Encapsulation efficiency (%)** | **Encapsulation efficiency (%)** | **Encapsulation efficiency (%)** | **Encapsulation efficiency (%)** |
| **1** | 8.842±1.440 | 6.016±10.421 | 4.139±5.587 | 44.400±23.716 |
| **2** | 21.477±6.986 | 16.904±3.859 | 7.103±6.205 | 3.631±4.778 |
| **3** | 5.084±2.045 | 30.563±12.643 | 12.149±1.134 | 13.101±11.348 |
| **4** | 26.231±1.911 | 18.161±9.467 | 13.904±12.087 | 21.830±18.959 |
| **5** | 22.889±1.719 | 26.268±5.109 | 2.955±2.727 | 6.366±4.327 |
| **6** | 15.028±13.039 | 49.696±17.840 | 28.160±15.096 | 37.487±29.611 |
| **7** | 33.019±1.756 | 4.271±7.397 | 10.580±9.173 | 22.351±3.006 |
| **8** | 4.170±3.812 | 1.324±2.293 | 20.507±10.937 | 55.533±20.583 |
| **9** | 4.694±2.537 | 24.208±16.210 | 27.769±1.908 | 5.735±9.933 |
| **10** | 2.575±4.460 | 0.706±1.223 | 15.999±16.458 | 57.417±4.525 |
| **11** | 27.443±14.471 | 1.823±1.595 | 19.097±1.312 | 39.337±17.456 |
| **12** | 18.725±1.820 | 1.001±1.733 | 19.907±1.116 | 21.880±9.068 |

**Supplementary Taleb 2.** Primer sequences.

| **Name** | **Forward primer (5’-3’)** | **Reverse Primer (5’-3’)** |
| --- | --- | --- |
| *Dectin-1*（Human） | GGAAGCAACACATTGGAGAATGG | AGAACCCCTGTGGTTTTGACA |
| *P-Selectin*（Mouse） | GAAAGGGCTGATTGTGACCCC | AGTAGTTCCGCACTGGGTACA |
| *β-actin*（Human） | CATGTACGTTGCTATCCAGGC | CTCCTTAATGTCACGCACGAT |
| *β-actin*（Mouse） | GGCTGTATTCCCCTCCATCG | CCAGTTGGTAACAATGCCATGT |

**Supplementary Taleb 3.** Blood glucose levels following administration of free, TAPE-encapsulated, and TAPE-released insulin in type I diabetic mice.

|  | **FUD-TAPE** | | | **INS** | | | **FUD-TAPE-INS** | | | **FUD-TAPE-INS-RE** | | |
| --- | --- | --- | --- | --- | --- | --- | --- | --- | --- | --- | --- | --- |
| 0 h | 20.3 | 30.8 | 20.6 | 25.4 | 19.0 | 16.9 | 33.3 | 25.3 | 21.8 | 17.6 | 17.5 | 16.8 |
| 0.5 h | 19.9 | 33.1 | 24.0 | 6.8 | 4.7 | 3.8 | 14.1 | 13.4 | 10.1 | 7.7 | 7.3 | 6.4 |
| 1 h | 22.2 | 32.2 | 26.1 | 2.1 | 2.4 | ＜1.1 | 6.4 | 4.5 | 3.7 | 4.9 | 4.4 | 3.5 |
| 1.5 h | 25.9 | 33.3 | 26.0 | ＜1.1 | 2.0 | ＜1.1 | 5.0 | 3.4 | 2.0 | 2.1 | 2.3 | 2.4 |

Blood glucose levels of type I diabetic mice at 0, 0.5, 1, and 1.5 h following administration of blank FUD-TAPE, free insulin (INS), insulin-loaded FUD-TAPE (FUD-TAPE-INS), or insulin recovered after release from FUD-TAPE (FUD-TAPE-INS-RE). For the FUD-TAPE-INS-RE group, insulin-loaded FUD-TAPE was incubated in PBS containing 10% BSA at 37 °C with sonication for 5 min, followed by centrifugation, and the insulin-containing supernatant was collected for administration. Values represent individual animals (n = 3 per group). Blood glucose concentrations are expressed as mmol L⁻¹.
